# Immunoglobulin Binding Protein 1 as a Potential Urine Biomarker in Patients with Lupus Nephritis

**DOI:** 10.3390/ijms20102606

**Published:** 2019-05-27

**Authors:** Eun-Ju Lee, Oh Chan Kwon, Byeongzu Ghang, Doo-Ho Lim, Do Hoon Kim, Seokchan Hong, Chang-Keun Lee, Bin Yoo, Yong-Gil Kim

**Affiliations:** 1 Division of Rheumatology, Department of Internal Medicine, University of Ulsan College of Medicine, Asan Medical Center, Seoul 05505, Korea; krys72@hanmail.net (E.-J.L.); alakzam41@naver.com (D.H.K.); medivineluke@gmail.com (S.H.); cklee@amc.seoul.kr (C.-K.L.); byoo@amc.seoul.kr (B.Y.); 2 Asan Institute for Life Science, Asan Medical Center, Seoul 05505, Korea; 3 Division of Rheumatology, Department of Internal Medicine, Yonsei University College of Medicine, Gangnam Severance Hospital, Seoul 06273, Korea; lunch0422@naver.com; 4 Division of Rheumatology, Department of Medicine, Jeju National University Hospital, Jeju 63241, Korea; indream81@naver.com; 5 Division of Rheumatology, Department of Internal Medicine, University of Ulsan College of Medicine, Ulsan University Hospital, Ulsan 44033, Korea; dlaengh@hanmail.net

**Keywords:** disease activity, immunoglobulin binding protein 1, inflammation, Lupus nephritis, renal tubular epithelial cells, urine biomarker

## Abstract

We evaluated the role of immunoglobulin binding protein 1 (IGBP1), a phosphoprotein associated with the B cell receptor (BCR) complex, as a urine biomarker in lupus nephritis (LN). The IGBP1 concentrations in plasma and urine of patients with LN, systemic lupus erythematosus (SLE) without nephritis and healthy controls were estimated by ELISA. IGBP1 expression in the kidneys of LN patients and transplantation donors was detected by immunohistochemistry. Microarray-based global gene expression profile of HK-2 cells with *IGBP1* knock-down and fluorescence-activated cell sorting (FACS) for intracellular IGBP1 expression in human peripheral blood mononuclear cells (PBMCs) was performed. Urine IGBP1 levels were elevated significantly in LN patients, and it correlated with the clinical activity indices (complement 3 (C3) level, anti-dsDNA antibodies titer, SLE Disease Activity Index-2000 (SLEDAI-2K) and histological activity index. IGBP1 expression was increased in LN patients as compared to the donors and was detected mainly in the tubules by histopathology. In microarray analysis, several genes related to SLE pathogenesis (*PPME1*, *ROCK2*, *VTCN1*, *IL-17R*, *NEU1*, *HLA-DM*, and *PTX3*) responded to siRNA-mediated *IGBP1* silencing. In FACS, IGBP1 was expressed mainly in the CD14^+^ cells. The overall expression of IGBP1 in PBMCs was higher in LN patients as compared with that in SLE patients without nephritis. Conclusively, urinary IGBP1 may be a novel biomarker reflecting the clinical and histological activities in LN.

## 1. Introduction

Systemic lupus erythematosus (SLE) is a complex autoimmune disease characterized by autoantibody production, immune complex deposition, and end-organ damage. Lupus nephritis (LN) is the most common and serious complication of SLE. Unfortunately, 10 to 15% of LN patients progress to end-stage renal disease, and the 5-year survival rate of LN patients is stalled at 82%, whereas the 5-year survival for SLE patients without nephritis is 92% [1].

Histological examination of the kidney is a valuable tool for the diagnosis, assessment, and prognostication of SLE patients. However, a kidney biopsy can be accompanied by significant morbidity and therefore, cannot usually be performed serially. A non-invasive, easily obtainable, and accurate marker that can be followed serially may, therefore, be of great value in monitoring LN patients [2,3]. Laboratory markers in current use, including serological determination of serum anti-double-stranded (ds)DNA antibodies and complement levels can be helpful clinically, however, the correlation between these and LN is weak [4].

Urine abnormalities and impairment of renal functions are the key manifestations of LN. Identification of specific biomarkers in LN, distinct from SLE patients without nephritis, is important for monitoring the disease activity and guiding treatment in LN. With respect to LN, urine biomarkers may be more specific for the diagnosis of kidney damage than serum biomarkers. Further, obtaining urine samples for laboratory testing is much easier and less invasive, making it an ideal biological sample for repetitive sampling in LN [5]. Presently, the hallmark of LN is considered to be proteinuria, and it is the principal urine biomarker that is estimated during screening and monitoring [6]. However, a consistent concordance between proteinuria and histological activity in LN patients is lacking [7]. Therefore, the potential role of several urinary biomarkers, such as VCAM-1, TNFR1, P-selectin, CXCL16, and TWEAK, reflecting renal activity in LN was examined [8,9,10]. However, these biomarkers are not validated for use in clinical settings [5].

Although several cell types could be dysregulated, B cells have emerged as central players in SLE and LN development, and play a role by secreting autoantibodies, presenting antigens to T cells, and secreting inflammatory cytokines [11,12,13]. In healthy individuals, B cells expressing autoreactive receptors are negatively selected during B cell maturation, but not in SLE and therefore, exert its pathogenic effects. B cells from SLE patients have an exaggerated B cell receptor (BCR) response along with receptor crosslinking, leading to increased tyrosine phosphorylation of the downstream signaling molecules [14]. In a recent genome wide association studies (GWAS), variants affecting B cell and pre–B cell signaling was found to affect both central and peripheral tolerance in SLE [15,16]. Therefore, B cell-targeting therapy in refractory LN produces therapeutic effects in SLE patients [17,18,19].

Immunoglobulin binding protein 1 (IGBP1) was originally discovered as a 52-kDa phosphoprotein associated with Ig-α in the BCR complex [20]. This protein interacts with the catalytic subunit of protein phosphatase (PP2A) and highly conserved serine/threonine phosphatase and regulates differentiation, proliferation, and apoptosis [21,22]. Several studies have reported that an abnormally high PP2Ac level alters the phenotype and functions of T cells by affecting the transcription factor activity including cAMP response element-binding protein, E74-like factor 1, and specificity protein 1 in SLE patients [23,24,25].

Therefore, there is a pressing need to find precise urinary biomarkers that reflect LN disease activity. Here, we evaluated whether IGBP1, a phosphoprotein BCR complex in the urine, is a potential biomarker representing LN activity clinically and histologically.

## 2. Results

### 2.1. Baseline Characteristics of the SLE Patients

The demographic characteristics and disease-related variables of the participants are presented in Table 1. The patients were predominantly female, and the mean ages were 39.4 (SLE without nephritis) and 39.1 (LN) years. The median disease duration of the patients without or with nephritis were 6.2 and 7.7 years, respectively. Compared to the SLE patients without nephritis, LN patients had higher levels of anti-dsDNA (*p* = 0.037) and SLE Disease Activity Index-2000 (SLEDAI-2K) (*p* < 0.001). The levels of complement 3 (C3), erythrocyte sedimentation rate (ESR), and C-reactive protein (CRP) did not differ in the SLE patients in both groups. With regard to concomitant medications, LN patients, but not SLE patients without nephritis, were treated with glucocorticoids (median, 15 mg/day), mycophenolate mofetil (30.8% patients), and cyclophosphamide (26.3% patients).

### 2.2. Urinary IGBP1 Level was Increased in Patients with Lupus Nephritis

**Figure 1:**
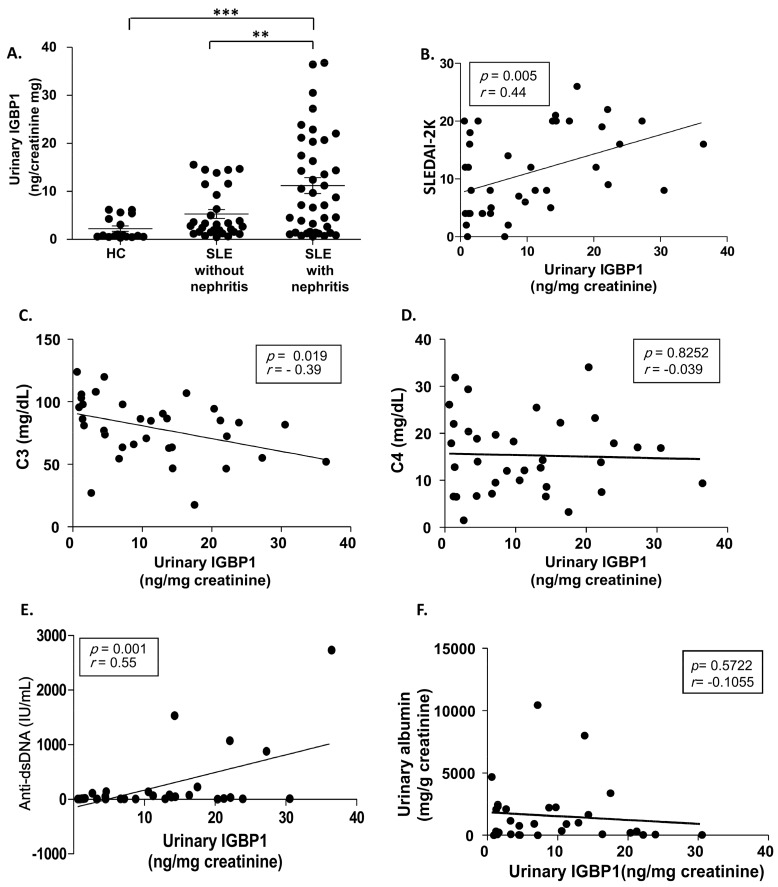
Urinary immunoglobulin binding protein 1 (IGBP1) levels in patients with LN. (**A**) Urinary IGBP1 levels; Correlation of urinary IGBP1 levels with Systemic Lupus Erythematosus Disease Activity Index 2000 (SLEDAI-2K) (**B**) complement 3 (C3) levels (**C**), complement 4 (C4) levels (**D**) anti-dsDNA levels (**E**), and albuminuria (**F**). ** *p* > 0.01; *** *p* > 0.001.

The levels of urinary IGBP1 were measured in SLE patients with (*n* = 39) and without (*n* = 30) nephritis, and healthy controls (*n* = 18) (Figure 1A). Urinary IGBP1 levels in LN patients were significantly higher than that in SLE patients without nephritis and healthy controls. Urinary IGBP1 levels in patients with LN showed a positive correlation with SLEDAI-2K and anti-dsDNA levels, and a negative correlation with C3 levels (Figure 1B,C,E). However, the levels were not associated with complement 4 (C4) levels and albuminuria (Figure 1D,F).

### 2.3. Tubular Expression of IGBP1 in Renal Pathology

**Figure 2:**
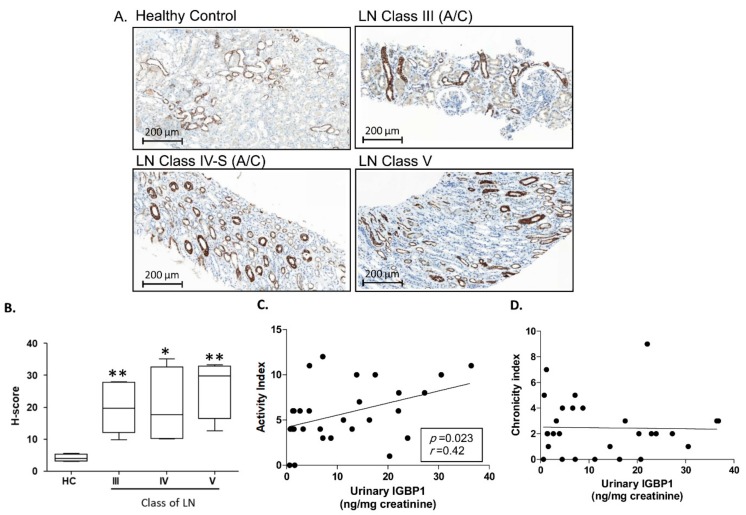
IGBP1 expression in the renal biopsy samples of patients with LN. (**A**) Immunohistochemical staining of IGBP1; (**B**) H-score of IGBP1 expression according to the class of LN; Correlation of urinary IGBP1 and histologic activity index (**C**) or chronicity index (**D**); * *p* > 0.05; ** *p* > 0.01.

The expression of IGBP1 in the renal tissues was investigated in 19 patients with LN and 5 kidney donors (healthy control) by immunohistochemistry. As shown in Figure 2A, strong expression of IGBP1 was observed mainly in tubular epithelial cells rather than the glomerular cells. In histological scoring, the patients with LN class III, IV, and V showed a higher expression of IGBP1 as compared to the healthy controls (Figure 2B). The levels of urine IGBP1 positively correlated with the histological activity index (Figure 2C) but not with the chronicity index (Figure 2D).

### 2.4. Microarray Analysis in IGBP1 siRNA Transfected HK-2 Cells

**Figure 3:**
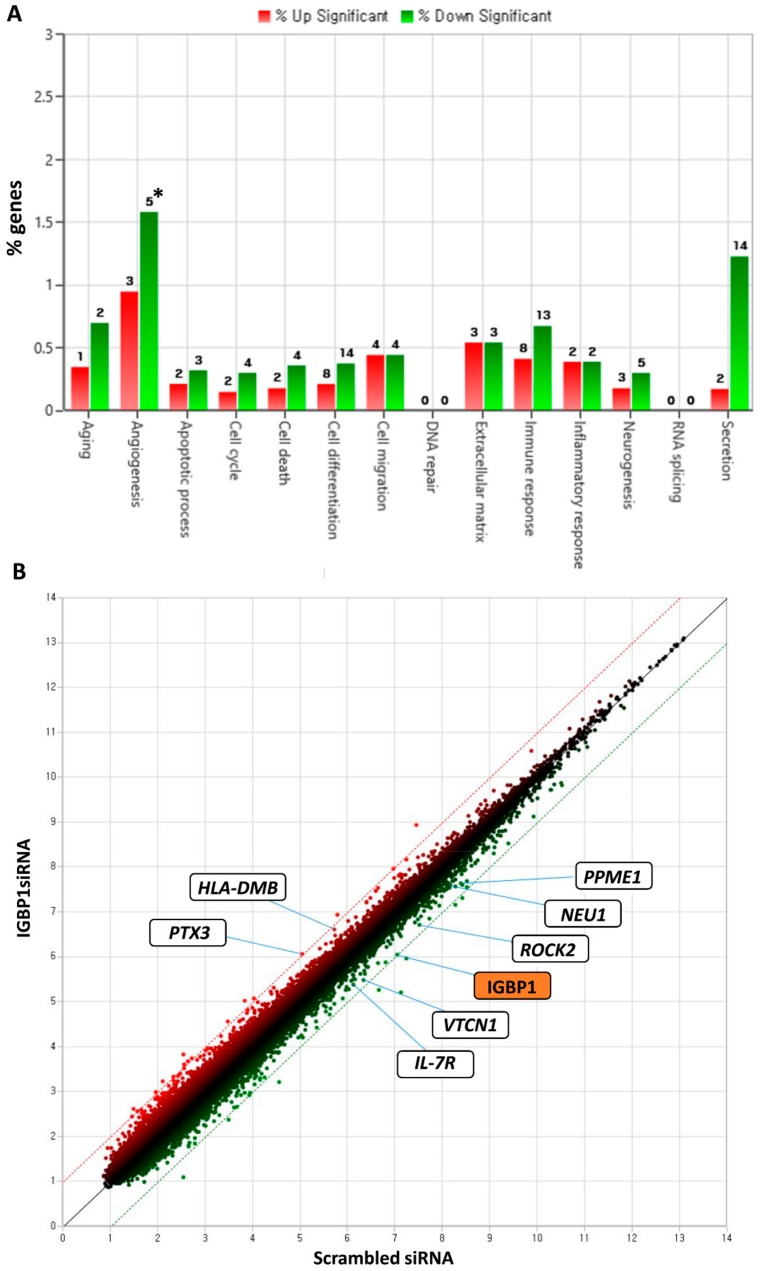
Transcripts regulated by IGBP1 knockdown in HK-2 cells. (**A**) Regulated biological pathways in IGBP1 siRNA transfected HK-2 cells; (**B**) Scatter plot of downregulated or upregulated transcripts in IGBP1 siRNA transfected HK-2 cells; *, number of genes.

To elucidate the function of IGBP1 in renal tubular epithelial cells, IGBP1 was silenced using siRNA in the human renal tubular epithelial cell line, HK-2 and microarray assay was performed and analyzed. A total of 88 and 104 transcripts met the filtering criteria (fold change value of >1.5 or ≤−1.5 and a *p*-value of < 0.05). Canonical pathway analysis using ingenuity pathway analysis was performed to identify the biological pathways that were significantly altered in IGBP siRNA- transfected cells. The altered pathways are shown in Figure 3A. Of the 192 transcripts, seven genes are known to be related to SLE pathogenesis (Figure 3B). These which included protein phosphatase methylesterase (PPME1), rho-associated, coiled-coil containing protein kinase 2 (ROCK2), B7 homolog 4 (B7-H4, coded by VTCN1), interleukin 7 receptor (IL-7R), and sialidase 1 (NEU1) were downregulated. The upregulated genes included major histocompatibility complex (MHC) class II, DM (HLA-DM), and Pentraxin 3 (PTX3).

### 2.5. Increased Plasma IGBP1 in SLE Patients and Distribution of IGBP1 in PBMCs

**Figure 4:**
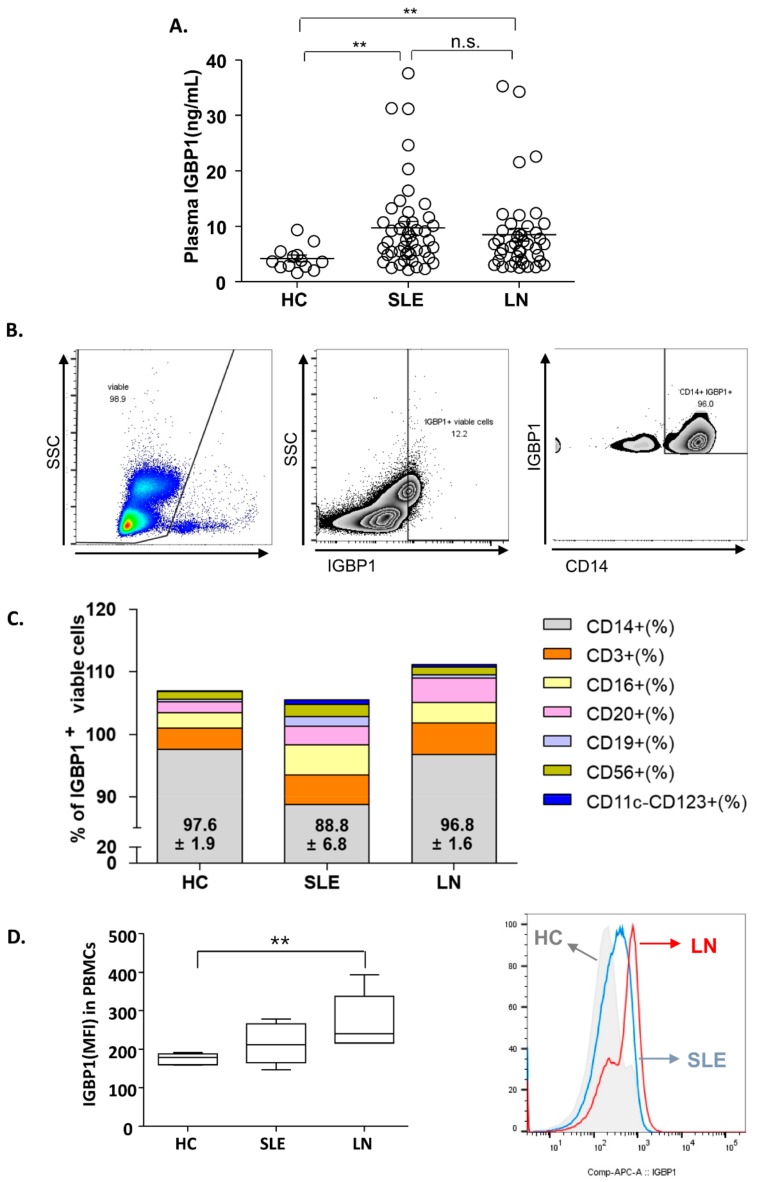
Plasma IGBP1 in SLE patients and distribution of IGBP1 in peripheral blood mononuclear cells (PBMCs). (**A**) plasma IGBP1 level; (**B**) IGBP1 expression in CD14^+^ cells in a patient with nephritis (representative); (**C**) Distribution of IGBP1 expression according to cell-type; (**D**) overall IGBP1 intensity in PBMCs; ** *p* > 0.01.

The levels of plasma IGBP1 in patients with SLE with (*n* = 39) or without (*n* = 30) nephritis and healthy controls (*n* = 18) were estimated (Figure 4A). The levels were increased in patients with SLE as compared to that in the healthy control. However, no significant difference was found in the plasma levels of IGBP1 between LN patients and patients with SLE without nephritis.

Analysis of the distribution of IGBP1 in PBMCs of healthy subjects showed that IGBP1 was mainly expressed in CD14^+^ cells, followed by CD3^+^, CD16^+^, and CD20^+^. In patients with SLE, the distribution of IGBP1 expression was similar to that of the healthy subjects or LN (Figure 4B,C). However, the overall intensity of IGBP1 in peripheral blood mononuclear cells (PBMCs) was increased in LN patients as compared to those with SLE without nephritis or healthy subjects (Figure 4D).

## 3. Discussion

In this study, we provide evidence for the potential use of IGBP1 as a biomarker in the urine of LN patients. This phosphoprotein of the BCR complex correlated with several indices including SLEDAI-2K, levels of C3 and anti-dsDNA antibodies titers suggesting SLE activity. Other researchers have demonstrated a 70% overlap between urine and kidney proteome [26,27], indicating that urine can better reflect the functions of the kidney than other body fluids.

Renal histological analysis showed that IGBP1 expression was predominant in tubular lesions, which correlated to the histological activity. However, urinary IGBP1 levels were not associated with the levels of albuminuria. Previous studies have suggested that the difference in urine protein types (albuminuria and non-albumin proteinuria) was useful in determining the origin of proteinuria in glomerular and tubulointerstitial diseases [28]. Moreover, non-albumin proteinuria was associated with severe tubulointerstitial inflammation in LN patients [29]. Taken together, urine IGBP1 probably originates in the tubulointerstitium rather than glomerulus, indicating that high levels of urine IGBP1 in LN patients might represent tubulointerstitial inflammation. Therefore, this study is noteworthy in that it suggests the pathogenic roles of IGBP1 on the renal tubular inflammation in LN patients.

Although LN classes were defined mainly by different glomerular changes, up to 70% of patients with active proliferative glomerulonephritis exhibit immunoglobulin deposition along the renal tubular basement membrane [30,31,32]. Further, the proximal tubular epithelial cells play a pivotal role in mediating pathological processes that affect long-term renal damages, including tubulointerstitial inflammation, epithelial-to-mesenchymal transition, and fibrosis [33,34,35]. Therefore, we evaluated the function of IGBP1 in human renal proximal tubular epithelial cells, HK-2. Gene silencing of *IGBP1* in HK-2 cells resulted in the upregulation of 88 genes and downregulation of 104 genes, some of which coded for proteins having roles in immune and inflammatory responses. Considering the interactions between the tubular epithelial cells and infiltrating T cells involved in tubular pathogenesis, we selected several IGBP1-associated molecules that were downregulated by siRNA-mediated silencing. *PPME1* catalyzes the demethylation of PP2A, which is highly expressed in T cells of SLE patients as compared to a healthy population and inhibits this enzyme by binding directly to the active site of PP2A [36]. *ROCK2*, an important regulator of T-cell effector function, is known to be activated by PP2A. As many as 60% of patients with SLE exhibit increased ROCK2 activity in their PBMCs [37]. *B7-H4* coded by *VTCN1* is a recently identified member of the B7 family. The soluble form of this costimulatory molecule is increased in the sera of SLE patients [38] and is detected only in the tubule epithelium of the renal tissues. It is also overexpressed in renal tissue in patients with serious tubular lesions [39]. *IL-7R* is a T- cell activation-related molecule and may function as a surrogate marker of LN activity [40]. Among the genes upregulated by siRNA-mediated silencing, *HLA-DM* plays a key role in MHC class II antigen presentation and CD4^+^ T- cell epitope selection [41]. Further, polymorphisms of *HLA-DM* alleles were found frequently in SLE patients [42,43].

Among the upregulated genes, *NEU1* codes for an enzyme that removes sialic acids from gangliosides and is highly expressed in kidneys [44]. Interestingly, blocking NEU inhibited IL-6 production in the mesangial cells of *MRL/lpr* lupus-prone mice [45]. *PTX3*, a long pentraxin having a role in the clearance of dying cells, modulates leukocyte recruitment and takes part in the resolution of muscle inflammation [46]. Hence, decreased levels of PTX3 could result in accumulation of cell debris and subsequent inflammation and autoimmunity. Consistent with this, Wirestam et al. [47] reported that serum PTX3 is markedly lower in SLE, particularly when IFN-α is detectable. Therefore, based on the inverse expression of *IGBP1* and *PTX3* in our microarray analysis, low PTX3 induced by high IGBP1 could be associated with the defective clearance of dying cells in SLE pathogenesis. Taken together, high levels of IGBP1 in kidneys of LN patients might activate several molecules associated with SLE pathogenesis leading to tubulointerstitial inflammation.

In a previous study [48], IGBP1 was shown to inhibit apoptosis, which suggests silencing *IGBP1* results in an upregulation of apoptotic genes. However, our microarray analysis showed several genes for apoptosis (*TNFSF10*) as well as anti-apoptosis (*SNAI2, EDN1* et al.) were upregulated by *IGBP1* deletion. Moreover, tubular atrophy, suggesting apoptosis, was not different in the tubules with and without IGBP1 expression, and histological chronicity index was also not associated with the level of IGBP1. Therefore, we could not assure the role of IGBP1 on apoptosis from the current data. Further study is needed to clarify profound phenotypes associated with apoptosis using *IGBP1* conditional knock-down animal.

Plasma IGBP1 levels were increased in patients with SLE but did not differ between LN and SLE without nephritis. Renal macrophage infiltration was reported as a strong prognostic biomarker for progression of LN [49], indicating that monocytes may have a potential role in renal damage in SLE. Interestingly, our fluorescence-activated cell sorting (FACS) analysis of IGBP1 distribution in PBMCs showed its expression mostly in CD14^+^ monocytes. Since the overall IGBP1^+^ expression was increased in LN patients, increased IGBP1^+^ PBMCs may be associated with LN development. Hence, long-term prospective studies are needed to elucidate the relationship between IGBP1^+^ PBMCs and IGBP1^+^ renal lesions in LN.

In conclusion, based on the present data, IGBP1 could be suggested as a protein involved in the pathogenesis of renal tubular inflammation in LN patients, and we demonstrated that the levels of urinary IGBP1 were higher in LN patients and the levels correlated positively with the clinical and histological feature. Furthermore, in LN patients, we recommend that estimating the level of urine IGBP1 will assist in identifying tubulointerstitial inflammation and thereby, can aid in deciding the course of further therapy.

## 4. Materials and Methods

### 4.1. Patients Selection and Estimation of IGBP1 Concentration

All SLE patients, who met the criteria of 1997 American College of Rheumatology classification [50], were recruited from the Rheumatology clinic, Asan Medical Center, from March 2014 to September 2014. LN was classified according to the criteria defined by the International Society of Nephrology/Renal Pathology Society in 2003 [51]. Histopathologic findings or the disease activity-related variables including CRP, ESR, anti-dsDNA antibodies, C3, complement 4 (C4), SLEDAI-2K, microscopic hematuria, and urine protein/creatinine ratio were extracted from the electronic medical records. The concentrations of urine IGBP1, adjusted by urine creatinine concentration, were estimated in the SLE patients with (*n* = 39) or without (*n* = 30) nephritis, and healthy subjects (*n* = 18) using a commercially available ELISA kit (USCN Life Science, Hankou, Wuhan, China). The kidney specimens were obtained at the time of renal biopsy from suspected LN patients or kidney transplantation donors. The Institutional Review Board of the Asan Medical Center in Seoul, South Korea, approved the study (IRB No. 2014-0568, 09 JUNE 2014). Written informed consent was obtained while collecting blood, urine, and tissue samples. 

### 4.2. Lupus Nephritis Activity and Chronicity Index Assessment

A histopathological activity index score ranging from 0 to 24, was assessed from six histological parameters including glomerular cell proliferation, fibrinoid necrosis or karyorrhexis, cellular crescents, hyaline thrombi or wire loop, glomerular leukocyte infiltration and interstitial inflammation [52]. The chronicity index score ranging from 1 to 12 was obtained by summing all the scores from each of the following parameters: glomerular sclerosis, crescent fibrous structure, tubular atrophy, and interstitial fibrosis [52]. A renal pathologist determined histopathological activity and chronicity index of the LN tissues.

### 4.3. Immunohistochemistry

The renal biopsy specimens were obtained from 17 patients with LN. The normal kidney specimens from kidney transplantation donors served as controls. The specimens were preserved in 10% buffered formalin, and 4 µm thick slices were obtained. These sections were stained using a BenchMark ULTRA automatic immunostaining device (Ventana Medical Systems, Tucson, AZ, USA) with OptiView DAB IHC Detection kit (Ventana Medical Systems) according to the manufacturer’s instructions. The histological grades of IGBP1 were analyzed by Vectra V 3.0 and inForm (Perkin Elmer, CA, USA).

### 4.4. Silencing IGBP1 in HK-2 Cells

HK-2 cells are human primary human proximal tubular epithelial cell (PTEC) immortalized by transduction with the human papilloma virus 16 *E6/E7* genes and share behavioral similarities with PTEC [53]. HK-2 cells (KTCC) were cultured in DMEM/F12 medium supplemented with 5% FBS. IGBP1 was silenced using small interfering RNA (siRNA). The siRNA duplex used in this study was designed to target the human IGBP1 sequence (HSS105247). Cells that achieved ≥ 80% confluence were transfected with IGBP1 siRNA or scrambled RNA (Thermo Fisher Scientific Inc., Rockford, IL, USA) using RNA MAXi transfection reagent (Thermo Fisher Scientific) according to the manufacturer’s instructions. After transfection for 48 h, the cells were harvested, and total RNA extraction was carried out for microarray analysis.

### 4.5. Microarray Analysis

#### 4.5.1. RNA Isolation and Gene Expression Profiling

In the present study, Affymetrix GeneChip^®^ Human Gene 2.0 ST Arrays were used for global gene expression analysis. The samples were prepared according to the instructions and recommendations provided by the manufacturer. Total RNA was isolated using RNeasy Mini Kit columns as described by the manufacturer (Qiagen, Hilden, Germany). The RNA quality was assessed by Agilent 2100 bioanalyzer using the RNA 6000 Nano Chip (Agilent Technologies, Santa Clara, CA, USA), and RNA was quantified by Nanodrop-1000 Spectrophotometer (Thermo Fisher Scientific). The RNA sample was used as input into the Affymetrix procedure as recommended by the protocol (http://www.affymetrix.com). Briefly, 300 ng of total RNA from each sample was converted to double-strand cDNA. Using a random hexamer incorporating a T7 promoter, amplified RNA (cRNA) was generated from the double-stranded cDNA template through an in vitro transcription reaction and purified with the Affymetrix sample cleanup module. cDNA was regenerated through random-primed reverse transcription using a dNTP mix containing dUTP. The cDNA was then fragmented by uracil-DNA-glycosylase (UDG) and apurinic/apyrimidinic (AP) endonuclease (APE 1) and end-labeled by terminal transferase reaction incorporating a biotinylated dideoxynucleotide. The fragmented end-labeled cDNA was hybridized to the GeneChip^®^ Human Gene 2.0 ST arrays for 17 h at 45 °C and 60 rpm as described in the Gene Chip Whole Transcript Sense Target Labeling Assay Manual (Affymetrix). After hybridization, the arrays were stained and washed in a Genechip Fluidics Station 450 (Affymetrix) and scanned by using a Genechip Array scanner 3000 7G (Affymetrix). The expression intensity data were extracted from the scanned images using Affymetrix Command Console software version 1.1 and stored as CEL files.

#### 4.5.2. Data Analysis

The intensity values of CEL files were normalized to remove bias between the arrays (M1), using the Robust Multi-array Average algorithm implemented in the Affymetrix Expression Console software (version 1.3.1.) (http://www.affymetrix.com). The whole normalized data were imported into the programming environment R (version 3.0.2), and the overall signal distributions of each array were compared by plotting using tools available from the Bioconductor Project (http://www.bioconductor.org) (M2) to check good normalization. After confirming whether the data were properly normalized, the differentially expressed genes (DEGs) that showed over the 2-fold difference between the average signal values of the control and treatment groups were selected manually. In addition, the normalized data of the selected DEGs were also imported into the R programming environment. After performing *t*-test, genes with a P-value < 0.05 were extracted as significant DEGs for further studies (M2). To classify the co-expression gene groups that had similar expression patterns, hierarchical clustering analysis was performed with the Multi Experiment Viewer software version 4.4 (http://www.tm4.org) (M3). Finally, using the web-based tool the Database for Annotation, Visualization, and Integrated Discovery (DAVID), the DEGs were functionally annotated and classified based on the information of gene function, such as OMIMDISEASE, GENE ONTOLOGY, KEGG PATHWAY and BIOCARTA databases to reveal the regulatory networks that they are involved in (http://david.abcc.ncifcrf.gov) (M4).

### 4.6. Surface and Intracellular Staining and Flow Cytometry

Fc receptors were blocked with Fcγ blocker (BioLegend, San Diego, CA, USA), and the surface markers were stained with BV450-conjugated anti-CD3 (BioLegend, clone:UCHT1), FITC-conjugated anti-CD19 (BioLegend, clone: HIB19), APC/Cy7-conjugated anti-CD20 (BioLegend, clone: 2H7), PE-conjugated anti-CD14 (BioLegend, clone: ΦM P9), PE-conjugated anti-CD11c (BioLegend, clone: B-ly6), PerCP/Cy5.5-conjugated anti-CD56 (BioLegend, clone: B159), FITC-conjugated anti-CD16 (BioLegend, clone:3G8), PE-conjugated anti-CD123 (BioLegend, clone: 6H6), and APC/Cy7-conjugated anti-CD4 (BioLegend, clone: RPA-T4). After fixing and permeabilization, IGBP1 was stained with Alexa 647-conjugated anti-IGBP1 (Novus Biologicals, Centennial, CO, USA).

### 4.7. Statistical Analyses

For comparison between two groups, *t*-test or Mann–Whitney *U* test was used for variables with normal distribution or non-normal distribution, respectively. Comparison among three groups was analyzed by one-way ANOVA or Kruskal–Wallis test for variables with normal distribution or non-normal distribution, respectively. To assess the correlation between urinary IGBP1 levels and clinical parameters, Spearman correlation analysis was used. The results were plotted with Prism 5.0. A P-value of < 0.05 was considered significant.

## Figures and Tables

**Table 1:** Baseline characteristics of the systemic lupus erythematosus (SLE) patients with and without nephritis.

	SLE without Nephritis (*n* = 30)	Lupus Nephritis (*n* = 39)	*p* Value
Female (N, %)	29 (96.7%)	38 (97.4%)	>0.999
Age (years, mean ± SD)	39.4 ± 8.5	39.1 ± 11.0	0.903
Disease duration (years, median, range)	6.2 (3.2–10.7)	7.7 (2.1–10.9)	0.410
Laboratory data			
Serum creatinine (mg/dl, median)	0.70 (0.63–0.80)	0.80 (0.70–1.10)	0.019
C3 (mg/dl, mean ± SD)	77.3 ± 24.3	81.3 ± 24.8	0.523
C4 (mg/dl, median)	11.7 (9.1–15.1)	14.0 (8.1–21.0)	0.037
Anti-dsDNA (IU/mL, median)	7.5 (5.3–20.0)	14.2 (7.7–78.8)	0.037
ESR (mm/hr, mean ± SD)	27.1 ± 13.8	33.2 ± 19.0	0.125
CRP (mg/dl, median)	0.10 (0.10–0.20)	0.11 (0.10–0.31)	0.129
Urine protein/creatinine ratio (mg/g, median)	NA	1009.5 (155.4–2275.6)	NA
Microscopic hematuria (N, %) ^#^	1 (3.3%)	19 (48.7%)	<0.001
Organ involvement (N, %)			
Renal	0 (0.0%)	39 (100.0%)	<0.001
Neurologic	2 (6.7%)	1 (2.6%)	0.576
Musculoskeletal	6 (20.0%)	8 (20.5%)	0.958
Mucocutaneous	4 (13.3%)	6 (15.4%)	>0.999
Serositis	2 (6.7%)	1 (2.6%)	0.576
SLEDAI-2K (mean ± SD)	4.48 ± 0.73	12.18 ±1.16	<0.001
Medications			
Glucocorticoids (mg/d, median) *	0.0 (0.0–10.0)	15.0 (6.3–20.0)	0.001
Hydroxychloroquine (N, %)	29 (96.7%)	31 (79.5%)	0.067
Mycophenolate mofetil (N, %)	0 (0.0%)	12 (30.8%)	0.001
Cyclophosphamide (N, %)	0 (0.0%)	10 (26.3%)	0.002
Azathioprine (N, %)	2 (6.7%)	7 (17.9%)	0.281
Methotrexate (N, %)	1 (3.3%)	0 (0.0%)	0.435

Abbreviations: SLE, systemic lupus erythematosus; ESR, erythrocyte sedimentation rate; CRP, C-reactive protein; NA, not available; SLEDAI-2K, Systemic Lupus Erythematosus Disease Activity Index 2000. ^#^ RBC ≥ 5/HPF (high power field), * Prednisolone equivalent.

## Abbreviations

BCR	B cell receptor
CRP	C-reactive protein
ESR	Erythrocyte sedimentation rate
LN	Lupus nephritis
MHC	Major histocompatibility complex
SLE	Systemic lupus erythematosus
